# Intravoxel incoherent motion (IVIM) MRI in pediatric patients with synovitis of the knee joint: a prospective pilot study

**DOI:** 10.1186/s12969-022-00756-w

**Published:** 2022-11-16

**Authors:** Britta Huch, Kilian Stumpf, Anna-Katinka Bracher, Volker Rasche, Daniel Vogele, Catharina Schütz, Aleš Janda, Meinrad Beer, Henning Neubauer

**Affiliations:** 1 Department of Diagnostic and Interventional Radiology, University Hospital Ulm, Albert-Einstein-Allee 23, 89081 Ulm, Germany; 2 Department of Internal Medicine II, Experimental Cardiovascular Imaging, University Hospital Ulm, Albert-Einstein-Allee 23, 89081 Ulm, Germany; 3 Department of Pediatrics, Medizinische Fakultät Carl Gustav Carus, Technische Universität Dresden, Dresden, Germany; 4 Department of Pediatric and Adolescent Medicine, University Hospital Ulm, Eythstrasse 24, 89075 Ulm, Germany; 5 SRH Clinic of Radiology, Albert-Schweitzer-Strasse 2, 98527 Suhl, Germany

**Keywords:** Diffusion-weighted imaging, Juvenile idiopathic arthritis -, Intravoxel incoherent motion - magnetic resonance imaging

## Abstract

**Background:**

Diagnosing synovial inflammation by administration of gadolinium-based contrast agents is limited by invasiveness and possible side effects, especially in children and adolescents.

**Purpose:**

We investigated diagnostic accuracy of diffusion-weighted (DWI) MRI with intravoxel incoherent motion (IVIM) imaging compared to contrast-enhanced MRI for detecting synovitis of the knee in a population of pediatrics and young adults. In addition we compared quantitative measures of synovial diffusion and perfusion to a group of healthy volunteers.

**Methods:**

In this prospective study, 8 pediatric patients with 10 symptomatic knees (6 girls and 2 boys, mean age 13 years) with known or suspected synovitis underwent pre- and post-contrast 3.0 T MRI of the knee joint and additional DWI sequences between October 2016 and July 2019. For comparison we enrolled 5 healthy young adults (2 women and 3 men, median age 27 years) with contrast-free MRI of both knees. Post-contrast T1w images and DWI images at b = 1000s/mm^2^ with apparent diffusion coefficient (ADC) maps of patients were separately rated by two independent and blinded readers with different levels of experience for the presence or absence and degree of synovitis along with the level of confidence. We measured signal intensity on DWI of synovium, joint effusion and muscle with regions of interests and calculated the IVIM-parameters tissue diffusion coefficient (D) and perfusion fraction (f) for patients and volunteers.

**Results:**

All patients showed at least some synovial contrast enhancement, 8 (80%) children knees were diagnosed with synovitis on contrast-enhanced (= ce)-T1w, the diagnostic standard. Ratings by the first and second reader on ce-T1w and DWI showed full agreement (kappa = 1) in diagnosing synovitis and substantial agreement (k = 0,655) for the degree of synovial enhancement. Interobserver agreement on DWI showed fair agreement (k = 0,220) between both readers. Diagnostic confidence was lower on DWI. Mean D- and f-values of muscle was comparable between patients and volunteers. Effusion mean D was higher, mean f was lower, synovial mean D was lower, mean f higher in patients than in volunteers. All differences were statistically significant (*p* < 0.001).

**Conclusions:**

Diffusion-weighted MRI with IVIM imaging remains a promising, though reader-dependent alternative to i.v. contrast-enhanced imaging in pediatric patients to reliably diagnose, or rule out, synovitis of the knee joint. We detected significantly restricted synovial diffusion and increased perfusion in patients compared to healthy volunteers.

**Trial registration:**

Ethical Comitee University Hospital Ulm, Nr. 320/16.

## Background

Joint inflammation is a clinical hallmark of septic arthritis (bacterial), reactive arthritis (bacterial and viral) and juvenile idiopathic arthritis (JIA). Knee synovitis is common in children and adolescents with JIA. JIA is defined as arthritis of unknown aetiology which has its onset before the 16th birthday and persists for at least 6 weeks [1]. As the synovial membrane is the primary site of inflammation [2], thickening of the synovial layer, synovial hyperperfusion and a varying degree of joint effusion are the hallmarks of arthritis on diagnostic imaging, in which magnetic resonance imaging (MRI) is more sensitive compared to physical examination [3]. Although synovitis of the knee is usually diagnosed in ultrasound examination in patients with known JIA, MRI is most helpful for imaging of large joints to detect non-rheumatological pathologies and monitor therapy response to anti-inflammatory treatment [4]. Diagnosing synovitis on MRI scans usually relies on intravenous application of gadolinium-based contrast agents for visualisation of inflamed synovial tissue [5]. The development of new, gadolinuim-free MRI scanning techniques has been a focus of ongoing research for the risk of allergic reaction, restricted use in case of impaired renal function, in light of reports on intracranial deposition of gadolinium [6] and concerns about long-term safety of MRI contrast agents, particularly in younger patients. Since 2009 Agrawal et al. [7] first described altered diffusivity and anisotropy in the inflamed synovia of adults with rheumatic arthritis, several studies reported further improvement of diffusion weighted imaging (DWI) techniques [8–12] for higher spatial resolution, better delineation of small anatomical structures and less artifacts [13–15] to examine inflammatory joint conditions, especially in children.

Advanced DWI techniques, such as intravoxel incoherent motion (IVIM) imaging [16] visualize both synovial diffusivity and perfusion. Indeed they may overcome the technical difficulties by making use of the so-called „T2w shine-through “artifact arising from joint effusion in the presence of small amounts of joint effusion. IVIM provides a valuable tool not only for the calculation of diffusion coefficients, but also the quantification of micro-vessel perfusion in tissues from multi-b-value DWI scans [17].

## Methods

### Study design and participants

In this prospective study we included eight consecutive children and young adults with overall ten knees and known or suspected synovitis of the knee joint who underwent routine diagnostic MRI between October 2016 and July 2019 at our institution. The patients were recruited at the department of Pediatric and Adolescent Medicine, consulting the Special Ambulance for Pediatric Rheumatology. Inclusion criteria were age between 0 till 25 years, consulting the Special Ambulance with suspected or having arthritis of the knee joint or follow up, indication for contrast-enhanced MRI and informed consent by them or their parents. Exclusion criteria were contraindication for 3 Tesla MRI – like implanted medical devices such as pace makers, neural stimulaters or insulin pumps, shell splinters, contraindication for application of constrast agent and missing informed consent. For comparison, we meanwhile enrolled 5 healthy young adults by posting in the University and University Hospital of Ulm who underwent contrast-free MR imaging of both knees. Inclusion criteria were age between 18 till 30 years, not having any known inflammatory pathologies of the knee joints and informed consent. Exclusion criteria were contraindication for 3 Tesla MRI – like implanted medical devices such as pace makers, neural stimulaters or insulin pumps, shell splinters and missing informed consent.

We conducted all study work in accordance with the Helsinki Declaration. The prospective study was approved by the institutional ethics committee (Nr. 320/16). Also, we obtained informed written consent from all participants or legal guardians for diagnostic procedures.

### MRI examination

All patients underwent clinical routine MRI on the same 3.0 Tesla scanner MAGNETOM Skyra (Siemens Healthcare, Erlangen, Germany). Standard sequences were scanned as needed for clinical diagnostic workup (e.g. T1-weighted turbo spin echo transverse, proton-density-weighted with fat saturated sagittal). Diffusion-weighted scans were acquired prior to injection of contrast agent using the following parameters: transverse segmented-readout multi-shot DWI (RESOLVE, Siemens Healthcare); repetition time (TR) = 3580 ms, echo time (TE) 1 = 57 ms, TE 2 = 88 ms; 5 readout segments; b-values 0/50/100/150/ 200/300/400/600/800/1000 s/mm^2^; fat suppression; trace-weighted; GeneRalized Autocalibrating Partial Acquisition (GRAPPA) with integrated Parallel Acquistion Techniques (iPAT) = 2; 3D Diagonal diffusion mode; monopolar gradient scheme; one average at b = 0, 50, 100, 150, 200, 300 s/mm^2^, two averages at b = 400 und 600 s/mm^2^, three averages at b = 800 und 1000 s/mm^2^; echo-planar imaging factor 63; voxel size 1.3 × 1.3 × 3.0 mm^3^; field of view 180 mm; acquisition time 5 min 56 s. Apparent diffusion coefficient (ADC)_0/1000_ maps were automatically calculated by mono-exponential curve-fitting on the MRI console. After intravenous injection of one weight-adapted standard dose of Gadolinium-based contrast agent (children under 18 years of age mostly goderate meglumine = Dotarem, Guerbet, Paris, France; over 18 years of age and one girl aged 11 years Gadovist, Bayer, Leverkusen, Germany), we acquired post-contrast transverse T1-weighted turbo spin echo with fat saturation, followed by coronal or sagittal T1-weighted scans when clinically indicated. Eight children/young adults and the five participants of the control group were examined in supine position feet-first and with a dedicated 15-channel transmit/receive knee coil of the manufacturer. In two girls (2 and 17 years old), both knees were examined in supine position head-first with an 18-channel body coil of the manufacturer in place, the first one following sedation administered by a paediatric anaesthesiologist.

### Intravoxel incoherent motion (IVIM) image processing

The IVIM model describes the total diffusion-weighted signal as a superposition of signal decay [16]. The perfusion-related signal contributes a fraction f to the unweighted (b = 0 s/mm^2^) signal S_0_ and decays exponentially with a so-called pseudo-diffusion coefficient Dp. In contrast, signal undergoing true diffusion is subject to exponential decay with the true diffusion coefficient D. IVIM describes the total diffusion-weighted signal intensity S as a bi-exponential function:

$$\textrm{S}={\textrm{S}}_0\left(\left(1-\textrm{f}\right)\ {\textrm{e}}^{-\textrm{bD}}+\textrm{f}\ {\textrm{e}}^{-\textrm{bDp}}\right)$$

Usually, the pseudo-diffusion coefficient Dp is approximately one order of magnitude larger than the tissue diffusion coefficient D. This means that the perfusion-related signal is more attenuated as compared to the diffusive signal for a given b value. At high b values (commonly ≥200 s/mm^2^) the perfusion-related signal is very low and may be neglected. In this case, the diffusion-weighted signal is described by:

$${\textrm{S}}_{\textrm{high}}={\textrm{S}}_0\left(1-\textrm{f}\right){\textrm{e}}^{-\textrm{bD}}$$

Fitting of the parameters D and f was done on a standard personal computer with custom written code in the software MATLAB® (version R2015b; MathWorks®, Natick, MA). Areas with signal intensity lower than 30% of maximum intensity on the unweighted (b = 0 s/mm^2^) images were masked out on the D and f parameter maps.

Parameter fitting was performed with the commonly used “segmented” two-step fit: first, D and f were calculated from images with b-values above a certain threshold b-value (here called b-threshold), where the influence of the pseudo-diffusion was negligible (assuming D* = 0), simplifying the fitting model to a mono-exponential signal decay function. Subsequently, these D- and f-values were used for the perfusion coefficient calculation in the bi-exponential model.

### Image analysis

**Fig. 1:**
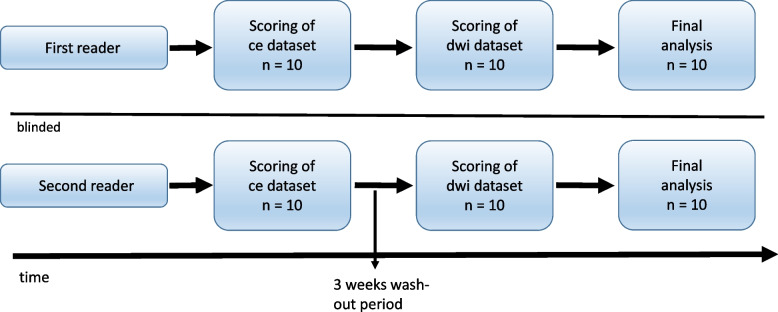
Flowchart of study patient knees examined independently by the first and second reader for qualitative image analysis. The scoring of the contrast-enhanced (= ce) and diffusion-wheighted (= dwi) images could be finally analyzed for all 10 patient knees

Qualitative image analysis was performed on a dedicated radiological workstation with commercially available PACS software (IMPAX EE R20.Ink, AGFA HealthCare, Mortsel, Belgium). A board-certified pediatric radiologist with 12 years of experience in pediatric extra-cranial DWI - also trained in this special DWI reading in knee joint synovium - (first reader) and a senior radiologist trained in pediatric imaging for 4 years (second reader) - but with no specific training in reading this DWI technique in joint synovium - reflecting different levels of experience, independently completed all qualitative image analysis for the patient group. Each reader was blinded to all clinical patient data and to the results of the other reader (see also Fig. 1). All contrast enhanced images and then three weeks later all diffusion-weighted images at b = 1000 s/mm^2^ with the corresponding ADC map in random order were assessed separately. Presence and degree of synovitis were recorded by each reader on a modified 5-item Likert scale, which is shown in Table 1. The categories 3 and 4 were considered to represent manifest synovitis/arthritis, while category 1 was thought to exclude the presence of manifest synovitis, based on imaging criteria. Category 2 indicated mild synovial irritation. The respective subjective level of diagnostic confidence (LoC) was documented along with each rating on a modified 3-item Likert-scale (1 = undecided, questionable, low confidence; 2 = intermediate, diagnosis established with some confidence; 3 = certain diagnosis, high confidence). The transverse contrast-enhanced T1w (ce-T1w) scan was considered the diagnostic standard of comparison.

**Table 1:** Image findings and degree of synovitis in qualitative analysis on contrast-enhanced and diffusion-weighted images

Category	Image findings	Degree of synovitis
0	non-diagnostic image quality	–
1	no signs of synovitis	No manifest synovitis
2	minimal circumscriptive or linear signal increase, not qualifying for synovitis	Mild synovial irritation
3	synovitis present, defined as marked linear signal increase of the synovial layer with < 2 mm in thickness	Manifest synovitis/arthritis
4	synovitis plus circumscriptive or diffuse synovial thickening ≥2 mm	Manifest synovitis/arthritis

Blinded to patient data and to the results of preceding qualitative image analysis, a resident radiologist with two years of experience in pediatric radiology performed quantitative analysis of patient and proband data using polygonal regions of interest (ROIs) manually and separately drawn on representative cross-section of diffusion-weighted images and the corresponding ADC map, which were placed side-by-side on the monitor. Quantitative data measured for synovium, joint effusion (if present) and muscle included signal intensity as the mean of two measurements on DWI b = 1000 s/mm^2^. Using custom-written code in the software MATLAB, the IVIM parameters perfusion fraction (f) and diffusion coefficient (D) were calculated from the total segmented tissue volume as mean value and standard deviation, minimum and maximum values. Quantitative analysis was repeated by the same reader after several weeks for assessment of intra-observer variability.

### Statistical analysis

Normally distributed data are presented as mean ± standard deviation. We used the Mann-Whitney test to compare means of two independent data sets deviating from normal distribution. Kappa statistics were calculated to assess inter-observer agreement. All data analysis were performed with SPSS Version 27 for Windows (IBM, Armonk, NY).

## Results

All MRI examinations were completed safely with no immediate adverse effects. All scans were consistently rated as diagnostic by both readers.

**Fig. 2:**
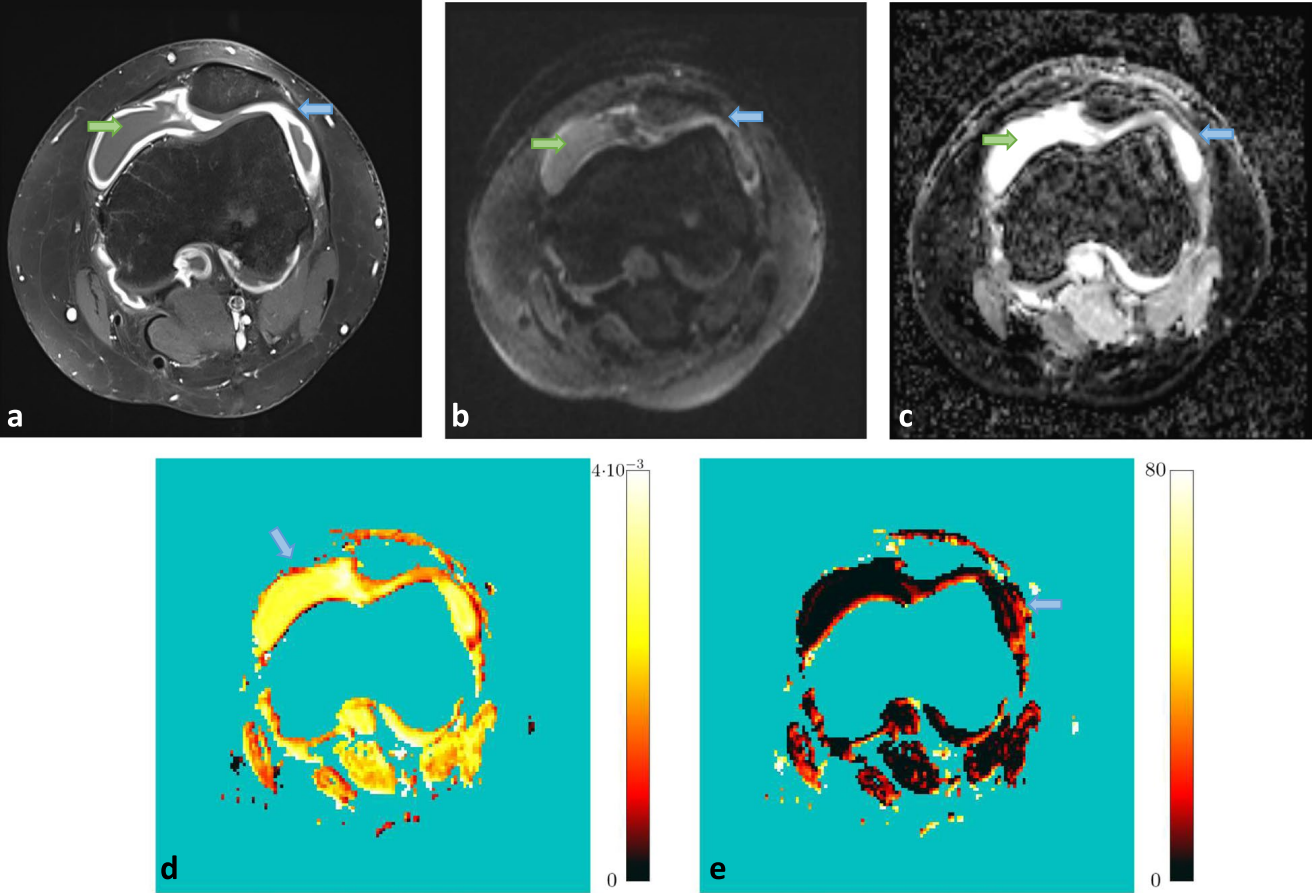
Persisting synovitis of the left knee joint under anti-inflammatory treatment in a 20 year-old male patient (patient 2 in Table 2) with known juvenile idiopathic arthritis (JIA). **a)** Contrast-enhanced T1-weighted MRI section showes diffuse synovial thickening and contrast-enhancement (blue arrow) along with marked joint effusion (green arrow), **b)** corresponding DWI at b = 1000 s/mm^2^ delineates high signal synovial layer (blue arrow) from low signal effusion (green arrow). **c)** ADC-map, **d)** masked D-map with colour-encoded diffusion ranging 0 - 4 × 10¯^3^ mm/s^2^ and **e)** f-map with colour-encoded perfusion fraction ranging 0 - 80% demonstrate restricted diffusivity and increased perfusion fraction of the synovial layer (blue arrows)

The patient group included eight consecutive children and young adults (female *n* = 6, male = 2) with overall ten knees and known or suspected synovitis of the knee joint. Median patient age was 13 years, ranging from 2 to 21 years. Five of them underwent imaging as diagnostic follow-up under anti-inflammatory treatment (methotrexate [MTX] *n* = 4) in juvenile idiopathic arthritis (JIA), one was newly diagnosed with JIA (no medication), one was later on diagnosed with tuberculoid Brodie-abscess and one with Lyme-arthritis. Further information to the patients with JIA (Number 1-5 and 8) is listed in Table 2. The other two patients had infectious arthritis. As an example, Fig. 2 shows images of one participant of the patient group with persisting synovitis under anti-inflammatory treatment. The volunteer group included 5 healthy young adults (female *n* = 2, male *n* = 3) with median age 27 years, ranging from 25 to 35 years, who underwent contrast-free MR imaging of both knees.

**Table 2:** Detailed information to the patients with diagnosed JIA

Patient Number	Age	Sex	Type of JIA	Age of initial diagnosis
1	21 years	Female	Polyarthritis	2 years
2	20 years	Male	Monarthritis	7 years
3	18 years	Female	Oligoarthritis	6 years
4	18 years	Female	Oligoarthritis	12 years
5	20 months	Female	Oligoarthritis	20 months
8	11 years	Female	Oligoarthritis	7 years

### Contrast-enhanced T1-weighted imaging as diagnostic standard

The reading completed by the first reader was considered the diagnostic standard of comparison. The first reader assigned patient knee to the categories of synovial signal as follows (see also Table 2): category 0 – no patients, all scans were of sufficient diagnostic quality; category 1 – 0 patients (0%) without any significant synovial contrast enhancement; category 2 – 2 patient knees (20%) with minimal circumscript or linear synovial contrast uptake, not qualifying for active synovitis; category 3 – 5 patient knees (50%) with marked synovial contrast uptake and mild synovial thickening not exceeding 2 mm; category 4 – 3 patient knees (30%) with strong synovial contrast uptake, marked synovial thickening and large joint effusion. Level of confidence (LoC) of the ratings by the first reader was high (LoC = 3) in 9 of 10 (90%) cases and medium (LoC = 2) in one (10%) case. All patients in category 3 and 4 showed varying degrees of joint effusion, one patient of category 2 had slightly increased intraarticular fluid. The patients were assigned to the categories of synovial signal as follows: category 2 – one patient with previously diagnosed JIA under anti-inflammatory treatment with MTX and one patient with synovitis by the cause of tuberculosis; category 3 – two patients with previously diagnosed JIA and persisting synovitis under anti-inflammatory treatment with MTX, one patient with newly diagnosed JIA and no medication, and one patient with previously diagnosed JIA and no anti-inflammatory treatment at the timepoint of examination; category 4 – two patients with previously diagnosed JIA with persisting synovitis under MTX, and one patient with newly diagnosed Lyme-arthritis. Based on these findings, the categories 1 & 2 and 3 & 4 were combined into a dichotomous variable, presenting 0 = no synovitis and 1 = active synovitis, for further analysis.

### Presence of synovitis on DWI

Compared to the diagnostic standard of contrast-enhanced MRI, the reading of DWI completed by the first reader showed consistent ratings in the four categories in 8 of 10 cases (80%) with a κ = 0.655 (*p* < 0.001), indicating substantial agreement across all categories based on the criteria by Landis and Koch [18]. The discordances occurred from downgrading on DWI, two cases of category 4 on contrast-enhanced MRI were rated as category 3 on DWI, so there was full agreement between the ratings with regard to presence (1) or absence (0) of active synovitis with a κ = 1 (p < 0.001). These downgrades were high confidence (LoC = 3) ratings on DWI. The overall diagnostic level of confidence was lower on DWI compared to contrast-enhanced MRI, including 5 (50%) ratings with high (LoC = 3), 4 (40%) ratings with intermediate (LoC = 2) and 1 (10%) rating with low (LoC = 1) confidence. The one low confidence rating pertained to choosing between category 1 and 2.

### Inter-observer agreement on contrast-enhanced imaging

Compared to the reference reading performed by the first reader on contrast-enhanced MRI, the readings of the second reader showed only slight agreement with κ = 0.048 (*p* < 0.001). Comparing the ratings of the second reader to the reference reading, there were three downgradings from category 4 to 3, each one downgrading from 3 to 2 and 2 to 1, and one upgrade from 3 to 4. All ratings by the second reader were done with high confidence. For the deciding on presence (1) or absence (0) of synovitis on contrast-enhanced MRI there was one disagreement between first and second reader and consecutive substantial agreement with κ = 0.737 (*p* < 0.001).

### Inter-observer agreement on DWI

Compared to the reference reading on contrast-enhanced MRI performed by the first reader, the DWI ratings of the second reader showed fair agreement with κ = 0.220 (p < 0.001). Comparing the DWI ratings of the second reader to reference, there was one up-grade from category 2 to 3 and one from 3 to 4, one down-grade from 4 to 3, one from 3 to 2 and one from 2 to 1. Three of the disagreements by the second reader were performed with high and two with intermediate confidence. Therefore the second reader considered each one patient without active synovitis (0) as having synovitis (1) and vice versa with resulting fair agreement and κ = 0.375 (*p* < 0.001) on DWI.

### Observer-dependent level of confidence

Diagnostic level of confidence (LoC) showed the following median values for ratings: median LoC = 3 on contrast-enhanced MRI for the first and second reader, median LoC 2.5 for DWI of the first and second reader. The proportion of high-confidence ratings (LoC = 3) on contrast-enhanced MRI was 90% for the first and 100% for the second reader, on DWI it was 50% for both the first and second reader.

### Quantitative analysis

In Table 3 there is a summary of quantitative image analysis. Mean tissue diffusion coefficient D and perfusion fraction f of muscle in patients were comparable to that in volunteers. Effusion mean D in patients was significant higher than in volunteers. In contrast there was significant lower mean effusion f in patients than in volunteers. For both mean D and f there was a significant difference for synovium values: in patients there were lower synovial perfusion coefficient D and higher perfusion fraction f as compared to volunteers. There was little overlap in signal of synovium of D-values in patients (range 1.51 to 2.27 10^− 3^ mm^2^/s) and in volunteers (range 2.21 to 2.94 10^− 3^ mm^2^/s), and no overlap of f-values (range 8.30 to 14.10% vs. 2.10 to 6.10%). We found moderate overlap in effusion D (1.87 to 3.32 10^− 3^ mm^2^/s vs. 1.66 to 2.32 10^− 3^ mm^2^/s) and no overlap in effusion f (1.20 to 5.90% vs. 6.40 to 22.30%).

**Table 3:** Results of quantitative analysis for mean tissue diffusion coefficient D (unit 10-3 mm2/s) and perfusion fraction f (unit %) for muscle, effusion and synovium in patients and volunteers. * = statistical significant difference (Mann-Whitney test *p* < 0.001)

		Patients	Volunteers
**Muscle**	Mean D (unit 10^−3^ mm^2^/s)	1.60 ± 0.14	1.70 ± 0.25 *
	Mean f (unit %)	6.90 ± 2.05	7.40 ± 2.60 *
**Effusion**	Mean D (unit 10^−3^ mm^2^/s)	2.43 ± 0.41	2.09 ± 0.44 *
	Mean f (unit %)	2.80 ± 1.30	13.20 ± 7.10 *
**Synovium**	Mean D (unit 10^− 3^ mm^2^/s)	1.81 ± 0.28	2.61 ± 0.34 *
	Mean f (unit %)	10.9 ± 2.50	4.40 ± 1.80 *

### Intra-observer variation of quantitative measurements

The between-measurements coefficient of variation for muscle mean D / f was 6.2% / 7.1% in patients and 8.7% / 18.9% in volunteers, for effusion mean D / f 9.4% /21.4% in patients and 7.4% / 31.8% in volunteers, for synovial mean D / f 3.8% / 2.7% in patients and 9.5% / 13.6% in volunteers.

## Discussion

To date, the gold standard for diagnosing synovitis on MRI requires intravenous administration of gadolinium-based contrast agents. Detecting synovial inflammation is crucial for patients with suspected rheumatoid arthritis or JIA. Only with a timely diagnosis, appropriate management and optimal monitoring, such chronic conditions may be well controlled. However, acquisition of contrast-enhanced MRI is limited by invasiveness of venipuncture and possible side effects of contrast agents. Research both in adults [6, 19, 20] and in children [21] has raised concerns with respect to accumulation of intracranial gadolinium deposition, although its clinical significance remains unclear. New, contrast-free, less invasive and less time-consuming image techniques should be available especially for children, to minimize adverse effects.

For a decade there have been advances in developing gadolinium-free synovia scanning techniques based on diffusion-weighted imaging. Unlike diffusion-weighted spin-echo sequences with non-segmented single-shot echo-planar imaging read-out, newer multi-shot DWI sequences (e.g. RESOLVE, Siemens), which scan in several steps or „segments“, have less artifacts and higher spatial resolution. In the present study voxel size decreased to 1.3 × 1.3 × 3.0 mm^3^ coming from 1.5 × 1.5 × 3 mm^3^ [15] and 1.8 × 1.8 x (4 to 6) mm^3^ [8, 12] in earlier studies. Introducing IVIM imaging as new DWI technique helps to better distinguish between high signal due to effusion and the inflamed synovium by visualizing both synovial diffusivity and synovial perfusion, and so to overcome the T2w shine-through artifact. This is important in cases with little or no synovitis but presence of small amounts of joint effusion, wether caused by other reasons, in early stages of arthritis or under anti-inflammatory treatment. In line with previous findings [22] this study data shows excellent agreement with a kappa value of 1 for diagnosing synovitis of the knee on DWI scans compared to contrast-enhanced T1w in the hands of an experienced observer. With substantial agreement and a kappa value of 0.655 in categorizing of synovitis by the first reader compared with the gold standard of contrast-enhanced MRI, there was only fair agreement between the DWI ratings of the 2nd reader compared to the gold standard. This further indicates, that experience and repetitive training on DWI reading are necessary for good diagnostic performance if using diffusion-weighted imaging alone in clinical routine. Compared to the ratings of contrast-enhanced images there was generally little lower level of confidence on DWI, maybe related to the still lower image contrast between synovium and effusion and less spatial resolution.

This is one of the first studies that compares quantitative data of DWI values between more or less affected knees with synovitis and unaffected healthy volunteers in a prospective setting. We detected significantly lower diffusion values in the synovium of patients than in that of healthy volunteers, indicating a pathologically restricted diffusion of water molecules for here being the site of inflammation. Consequently, we observed significantly higher synovial perfusion values in patients than in volunteers due to increased mircoperfusion of the synovial layer. The number of patients included in this proof-of principle-study was not sufficient to search for differences in synovial diffusivity and perfusion between different degrees of synovitis. We are going to address this question in the course of the on-going study.

For the knees of the volunteers naturally having no or only slightly increased intraarticular fluid, the direct comparison of effusion values between the groups is somewhat critical, although there was significant difference in both D and f. For physiological explanation, perfusion fraction f in effusion should be expected to be low to zero because there are no vessels in the joint fluid. Already in earlier studies [17] we observed non-zero values for effusion f in affected knees, which can be explained by signal noise and partial volume effects. Trying to measure f in the - if present - often only slightly increased synovial fluid of healthy volunteers, we found even higher values. This is mostly due to an amplification of the partial volume effect, for the small amount of effusion is immediatly adjacent to the synovium with higher values. Mean D- and f-values fell in range with previous work on children with JIA and at 1.5 T [17], especially for muscle tissue with mean D 1,65 ± 0,13 10^− 3^ mm^2^/s (1.64 ± 0.09 10^− 3^ mm^2^/s [16]) and mean f 7.2 ± 1.9% (9.6 ± 1.8% [16]) and synovium with mean D 1.78 ± 0.25 10^− 3^ mm^2^/s (1.95 ± 0.31 10^− 3^ mm^2^/s [16]) and mean f 10.8 ± 2.3% (8.1 ± 2.4% [16]). Therefore muscle tissue remains a good internal reference standard for ADC, as large and rather homogenous tissue volumes with little partial volume effects and low between measurement variation can be measured [15]. Nevertheless, there will be always some variation in quantitative parameters when comparing diffusivity data across different studies and research groups in respect to different hardware and software setups as well as individual factors like slightly altered placing of ROIs.

In this study we abstained from adopting the JAMRIS-score as validated MRI scoring system for assessing disease activity in children with JIA [5] for several reasons. We had a mixed patient population with synovitis of the knee for also other reasons, like infectious conditions. JAMRIS score focusses on synovial morphology only in terms of hypertrophy and needs further accommodation to functional parameters of disease activity, as for example parameters derived from IVIM modelling, diffusivity or dynamics of synovial contrast enhancement to be more sensitive to changes in disease activity and be a better indicator for therapy response [17].

The main methodological limitation of our prospective study was the limited number of participants in this proof-of-principle approach. Due to the small number of patients, and within them some young adults, we had a distortion of the median age (13 years), children with JIA often are younger. We could only recruit few patients, because we often encounter preexisting MRIs when presenting first at our institution, follow-ups were normally done via ultrasound. Another limitation lies in comparison of quantitative measurements between children and adolescents as patients on the one hand with adults as healthy volunteers on the other hand. For ethical reasons, acquisition of contrast-enhanced images in healthy paediatric volunteers is not feasable.

One problem we encountered was the only substantial agreement between the two readers when diagnosing or ruling out synovitis on contrast-enhanced MR images. In one case the second reader diagnosed synovitis although reference reading rated no synovitis. This is most probably due to a lack of specific training of the second reader, the first reader was familiar with this method for many years. So we want to emphasize the importance of training in this special reading of extracranial DWI in joint synovia, maybe with standardized analyzing protocols and in future the help of Artificial Intelligence.

Strengths of the study include that the same 3.0 T scanner was used for patients and healthy controls, that the technique of IVIM was compared with the diagnostic standard of contrast-enhanced MRI, we had a control group of healthy volunteers, the two readers were independent and blinded, and the first reader was a specialized Pediatric Radiologist and familiar to that kind of DWI reading.

With DWI imaging as a non-invasive and safe scanning technique, nearly free of adverse effects, there can be established further prospective cohort studies with both symptomatic and asymptomatic patients and repeated follow-up scans to get significant data. With the help of advanced imaging techniques, spatial resolution is improved but still limited in regard to thickness of the synovial layer, especially in very young children and at early stages of the disease. So there is need to get even better resolution on DWI images to get near to the excellent discrimination of anatomical structures on contrast-enhances MRI to date.

## Conclusions

Diffusion-weighted MRI with IVIM imaging presents a promising, though reader-dependent alternative to i.v. contrast-enhanced imaging in pediatric patients to reliably diagnose, or rule out, synovitis of the knee joint. We detected significantly restricted synovial diffusion and increased perfusion in patients with suspected synovitis or synovitis, compared to healthy volunteers.

## Data Availability

The datasets generated and analysed during this study are available from the corresponding author on reasonable request.

## Abbreviations

ADC: Apparent diffusion coefficient
D: Diffusion coefficient
Dp: Pseudo-diffusion coefficient
DWI: Diffusion-weighted imaging
e.g.: Example given
f: Perfusion fraction
GRAPPA: GeneRalized Autocalibrating Partial Acquisition
iPAT: Integrated Parallel Acquistion Techniques
i.v.: Intravenous
IVIM: Intravoxel incoherent motion
JIA: Juvenile idiopathic arthritis
LoC: Level of confidence
mm: Millimeter
MTX: Methotrexate
MRI: Magnetic resonance imaging
Nr.: Number
RESOLVE: Proper name of DWI sequence by Siemens Healthcare
ROI: Region of interest
S: Signal intensity
s: Second
TE: Echo time
TR: Repetition time
w: Weighted
